# ERβ decreases the invasiveness of triple-negative breast cancer cells by regulating mutant p53 oncogenic function

**DOI:** 10.18632/oncotarget.7300

**Published:** 2016-02-10

**Authors:** Igor Bado, Fotis Nikolos, Gayani Rajapaksa, Jan-Åke Gustafsson, Christoforos Thomas

**Affiliations:** ^1^ Department of Biology and Biochemistry, Center for Nuclear Receptors and Cell Signaling, University of Houston, Houston, Texas 77204, USA

**Keywords:** estrogen receptor β, mutant p53, triple-negative breast cancer, cell invasion, p63

## Abstract

Most (80%) of the triple-negative breast cancers (TNBCs) express mutant p53 proteins that acquire oncogenic activities including promoting metastasis. We previously showed that wild-type ERβ (ERβ1) impedes epithelial to mesenchymal transition (EMT) and decreases the invasiveness of TNBC cells. In the present study we searched for signaling pathways that ERβ1 uses to inhibit EMT and invasion in TNBC cells. We show that ERβ1 binds to and opposes the transcriptional activity of mutant p53 at the promoters of genes that regulate metastasis. p63 that transcriptionally cooperates with mutant p53 also binds to ERβ1. Downregulation of p63 represses the epithelial phenotype of ERβ1-expressing cells and alters the expression of mutant p53 target genes. These results describe a novel mechanism through which ERβ1 can disturb oncogenic signals to inhibit aggressiveness in TNBCs.

## INTRODUCTION

Of all breast cancers, clinical management of the basal-like subtype is particularly challenging due to lack of effective targeted therapies. Most of basal-like tumors are typically negative for estrogen receptor α (ERα), progesterone receptor and human epidermal growth factor receptor (HER)-2 and often referred to as triple-negative breast cancers (TNBCs). Basal-like cancers occur more frequently in younger women, they are biologically aggressive and often develop distant metastases [1, 2]. Most (80%) of these highly aggressive tumors harbor mutations in *p53* gene [1]. The majority of these mutations result in the expression of a protein with single amino acid substitutions in the DNA-binding domain (DBD) [3]. Because of alterations in the DNA-binding activity or the structure of the DBD, mutant p53 proteins either lose the tumor suppressor activity or acquire oncogenic function. Tissue culture and animal-based studies have demonstrated that mutant p53 proteins gain oncogenic properties that are independent of loss of wild-type p53 function. Expression of mutant p53 in p53 null cell lines promotes proliferation and invasion [4]. In mice harboring tumor-associated p53 mutations there is development of more invasive and metastatic tumors than in p53 null mice [5, 6].

All p53 family members exist as N-terminal variants derived from alternative promoter transcription (full length (TA) and truncated (ΔN)) and C-terminal isoforms (α, β, γ) produced by alternative splicing in the C-terminus. Interactions between the same or different family members represent one of the mechanisms that regulate their activity [7–9]. Only p53 with point mutations in the DNA binding domain that alter its conformation can interact with p63 and p73. TAp63 regulates gene expression to decrease the activity of cell surface receptors including EGFR and cell invasion [10–13]. By binding to p63 and preventing its normal transcriptional activity, mutant p53 promotes cell invasion [10, 12, 14, 15]. Although mutant p53 retains some DNA binding activity, it tethers to specific DNA sequences through other transcription factors including p63. This may account for the shared mutant p53 and p63 target genes that were identified in cancer cells [16]. Other mutant p53-interacting proteins that alter its gain-of-function include MDM2, PIN1, ANKRD11 and SMAD2 [7, 17, 18].

Another regulator of p53 is estrogen. Estrogen signaling is mediated through two estrogen receptor (ER) subtypes, ERα and ERβ. ERα is the principal biomarker for directing endocrine therapies and the primary therapeutic target in breast cancer. Wild-type ERβ (ERβ1) correlates with better survival in patients with TNBC [10, 19–21]. Interestingly, ERs have been shown to alter wild-type and mutant p53 transactivation. They transcriptionally cooperate with p53 through two mechanisms. One functions when ERs and p53 bind to their cognate response elements without a physical interaction [22] and the other requires binding of ERα to wild-type p53 which results in repression of p53 function [23–25]. In contrast to ERα, the interaction between ERβ and p53 and its effects on transcription have not been studied and is the subject of the present study. We, and others, have previously shown that ERβ1 impedes epithelial to mesenchymal transition (EMT) and decreases the invasiveness of mutant p53 TNBC cells by repressing EGFR signaling [26, 27]. However, the mechanism underlying the association of ERβ1 with the decreased EGFR activity and cell invasion has remained elusive. In the present study, we demonstrate the inhibition of mutant p53 oncogenic function as one of the mechanisms employed by ERβ1 to decrease invasion in TNBC cells.

## RESULTS

### Anti-migratory activity of ERβ1 correlates with inhibition of mutant p53 function

**Figure 1:**
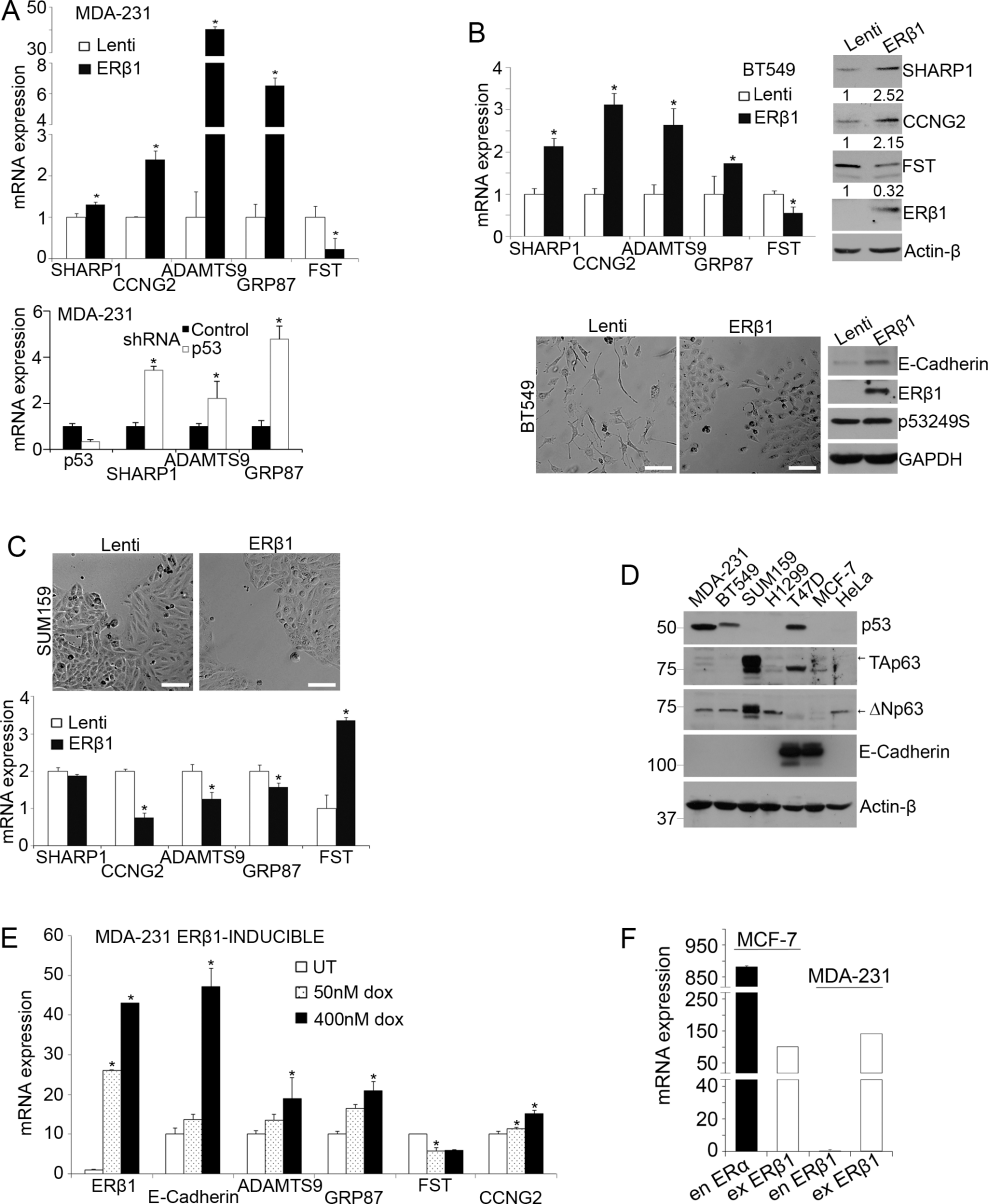
ERβ1 regulates mutant p53 target genes (**A**–**C**) TNBC cells were stably infected with lentivirus containing an empty vector (Lenti) or pLenti-FLAG-ERβ1 plasmid (ERβ1). (A) Top: The mRNA levels of mutant p53 target genes were quantified in control (Lenti) and ERβ1-expressing MDA-MB-231 (MDA-231) cells by real-time PCR and normalized to control cells (*Follistatin*, FST). Values are mean ± standard deviation (S.D.) of three independent experiments; *P ≤ 0.05. Bottom: mRNA levels of mutant p53 target genes in MDA-MB-231 cells after transfection with p53shRNA. (B) Top: mRNA and protein levels of mutant p53 target genes in control and ERβ1-expressing BT549 cells. Band intensities were analyzed by densitometry and normalized to Actin-β. The numbers under each immunoblot show the fold change compared to the control cells and represent the median of three experiments. Bottom: Morphology (left) and protein levels of ERβ1, mutant p53249S and E-cadherin (right) in control and ERβ1-expressing BT549 cells (scale bars, 100 μm). (C) Morphology (top) and mRNA levels of mutant p53 target genes (bottom) of control and ERβ1-expressing p53 null SUM159 cells. Values represent the mean ± S.D. of three independent experiments; *P ≤ 0.05. (**D**) Protein levels of p53, TAp63, ΔNp63 and E-cadherin in wild-type cell lines. (**E**) MDA-MB-231 cells were stably infected with lentivirus containing the pINDUCER-FLAG-ERβ1 plasmid and left untreated (UT) or treated with 50 nM or 400 nM doxycycline for 24 h for gradual induction of ERβ1 expression. Graph indicates the mean of three experiments; *P ≤ 0.05. (**F**) mRNA levels of exogenous (ex) ERβ1 in MCF-7 and MDA-MB-231 cells are compared with those of endogenous (en) ERα in MCF-7 cells. Graph shows the mean of three independent experiments.

In the present study we searched for ERβ1-interacting proteins and target genes that may account for the decreased invasiveness of ERβ1-expressing TNBC cells [26, 27]. We focused on mutant p53 signaling since *p53* is frequently mutated in TNBC and mutant p53 proteins promote tumor metastasis [10, 12, 17, 28]. We used as an indicator of mutant p53 gain-of-function the expression of genes that are regulated by mutant p53. We focused on those genes that inhibit metastasis in breast cancer including *SHARP-1* and the ERα-regulated *CCNG2* [3, 10, 29–31] and the pro-metastatic factor *Follistatin* [32]. As shown in Figure 1A (top), expression of ERβ1 in mutant p53 (p53280K)-expressing MDA-MB-231 cells upregulated *SHARP-1*, *CCNG2* and the tumor suppressor *ADAMTS9* [33] and downregulated *Follistatin*. The relevance of mutant p53 to the expression of these genes was further demonstrated by the upregulation of *SHARP1*, *ADAMTS9* and *GRP87* following knockdown of mutant p53 in MDA-MB-231 cells (Figure 1A, bottom). A similar gene expression signature was observed following upregulation of ERβ1 in another TNBC cell line. BT549 cells have mesenchymal-like morphology and express a different hot spot p53 mutant (p53249S). The changes in the expression of *SHARP-1*, *CCNG2* and *Follistatin* mRNAs were also confirmed at the protein level (Figure 1B, top). In addition to altering the expression of metastasis-associated genes, ERβ1 induced epithelial transformation in these cells as it did in MDA-MB-231 cells (Figure 1B, bottom) [27]. In contrast to mutant p53-expressing TNBC cells, ERβ1 did not alter the epithelial-like morphology of the p53 null SUM159 TNBC cells (Figure 1C, top). In these cells, ERβ1 was found to regulate the expression of *Follistatin*, *ADAMTS9* and *CCNG2* in opposite direction compared with the mutant p53-expressing cells (Figure 1C, bottom). This may be associated with a different mode of gene regulation in ERβ1-expressing cells that lack p53 and may depend on p63 isoforms that target the same group of genes and show unique expression in these cells (Figure 1D) [10]. We also analyzed the same genes in MDA-MB-231 cells with differential expression of ERβ1. Gradual upregulation of ERβ1 resulted in a progressive increase of *ADAMTS9*, *GRP87* and *CCNG2* and decrease of *Follistatin* levels (Figure 1E and Supplementary Figure 1). We previously showed that the levels of the transfected ERβ1 in TNBC cells are comparable with those of the endogenous receptor in MCF-7 cells [27]. In the present study, we compared the levels of the transfected ERβ1 in TNBC cells with the expression of endogenous ERα in MCF-7 cells, an indicator of biologically relevant ER expression in breast cancer cells. As shown in Figure 1F, the transfected ERβ1 in TNBC cells is expressed at lower levels compared with the endogenous ERα in MCF-7 cells. Despite an expected variation in the expression due to transfection, these results indicate the relevance of the ERβ1 expression system to human breast cancer cells.

**Figure 2:**
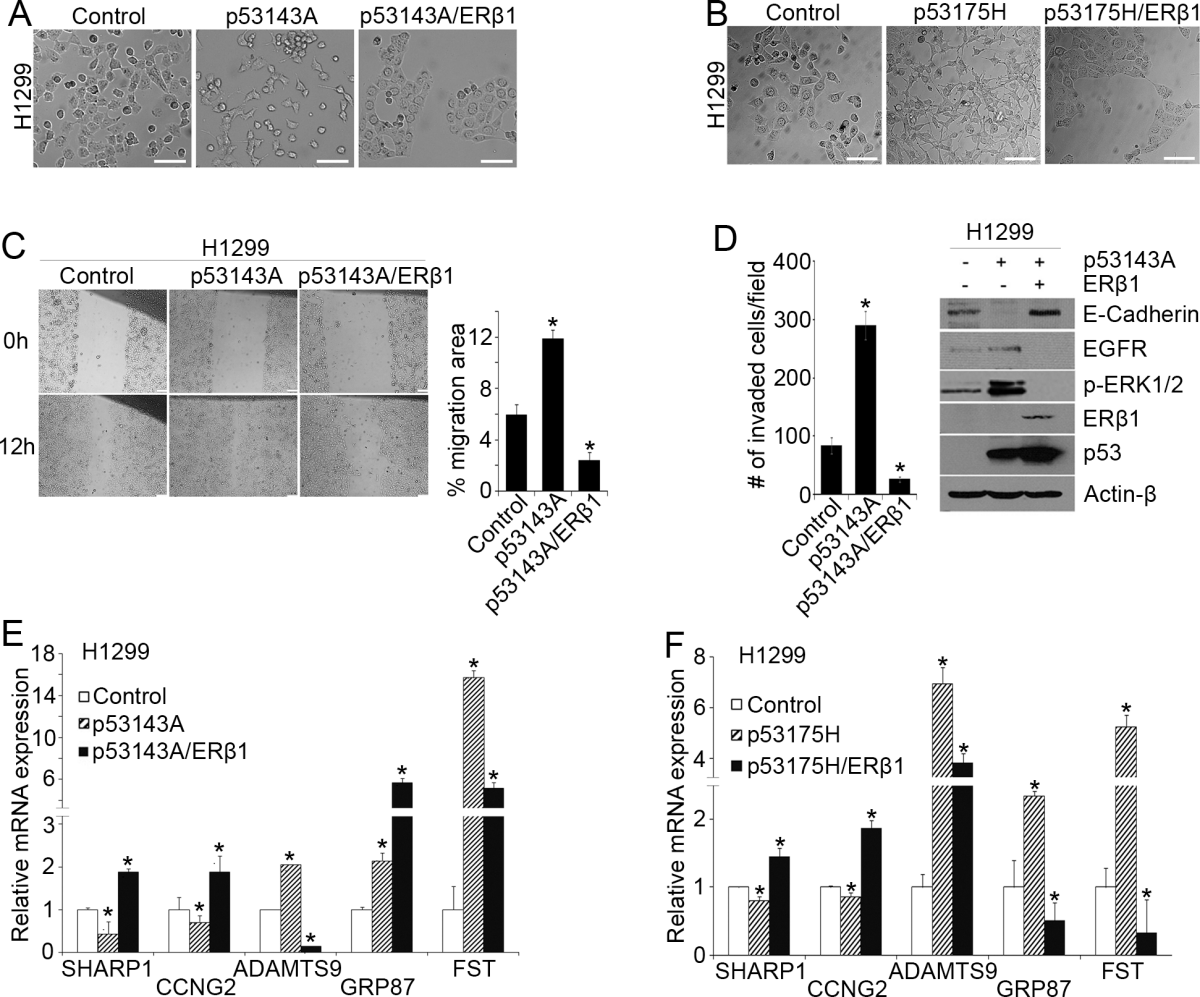
ERβ1 decreases cell invasion by regulating mutant p53 target genes (**A**–**B**) H1299 cells after stable transfection with empty vectors (control), or recombinant mutant p53143A or p53143A and ERβ1 plasmids (A) as well as mutant p53175H or p53175H and ERβ1 plasmids (B) (scale bars, 100 μm). (**C**) Left: Migration in control, p53143A- and p53143A/ERβ1-expressing H1299 pooled colony cells was assessed with wound-healing assay. Right: The area of cell migration in the wound was measured at the time of scratching the cell monolayer and 12 h later using ImageJ software. Values represent the mean ± S.D. of fold changes in migration area (12 h/0 h) from three experiments; *P ≤ 0.05. (**D**) Left: Invasion was assessed in control, p53143A- and p53143A/ERβ1-expressing H1299 cells with matrigel-coated Transwell chambers. The cells that invaded were quantified in five independent fields. The graph indicates the mean (cell number per field) of three experiments; *P ≤ 0.05 indicated. Right: E-cadherin, EGFR and phospho-ERK1/2 levels in control, p53143A- or p53143A/ERβ1-expressing H1299 cells. (**E**–**F**) mRNA levels of mutant p53 target genes in control, p53143A-, p53143A/ERβ1-, p53175H- and p53175H/ERβ1-expressing H1299 cells (*Follistatin*, FST).

To corroborate our findings, we examined the effects of ERβ1 upregulation on mutant p53 function in H1299 lung cancer cells. These cells are null for p53 and undergo mesenchymal reprogramming after mutant p53 expression [10, 12, 34]. We established pooled colony cell lines that express the frequently altered in human cancers p53 gain-of-function mutants p53143A and p53175H alone or together with ERβ1 [35, 36]. Upregulation of ERβ1 reversed the mutant p53-induced mesenchymal-like phenotype (Figure 2A and 2B), the increase in cell migration and invasion (Figure 2C and 2D, left) [10, 12], the downregulation of the epithelial marker E-cadherin and upregulation of EGFR signaling (Figure 2D, right). In addition, expression of ERβ1 reversed the downregulation of *SHARP-1* and *CCNG2* and upregulation of *Follistatin*. A difference in the expression pattern of *ADAMTS9* and *GRP87* may be associated with a different function of p53 mutants in the presence of varying expression of TA and ΔNp63 (Figure 2E and 2F).

### ERβ1 interacts with mutant p53

**Figure 3:**
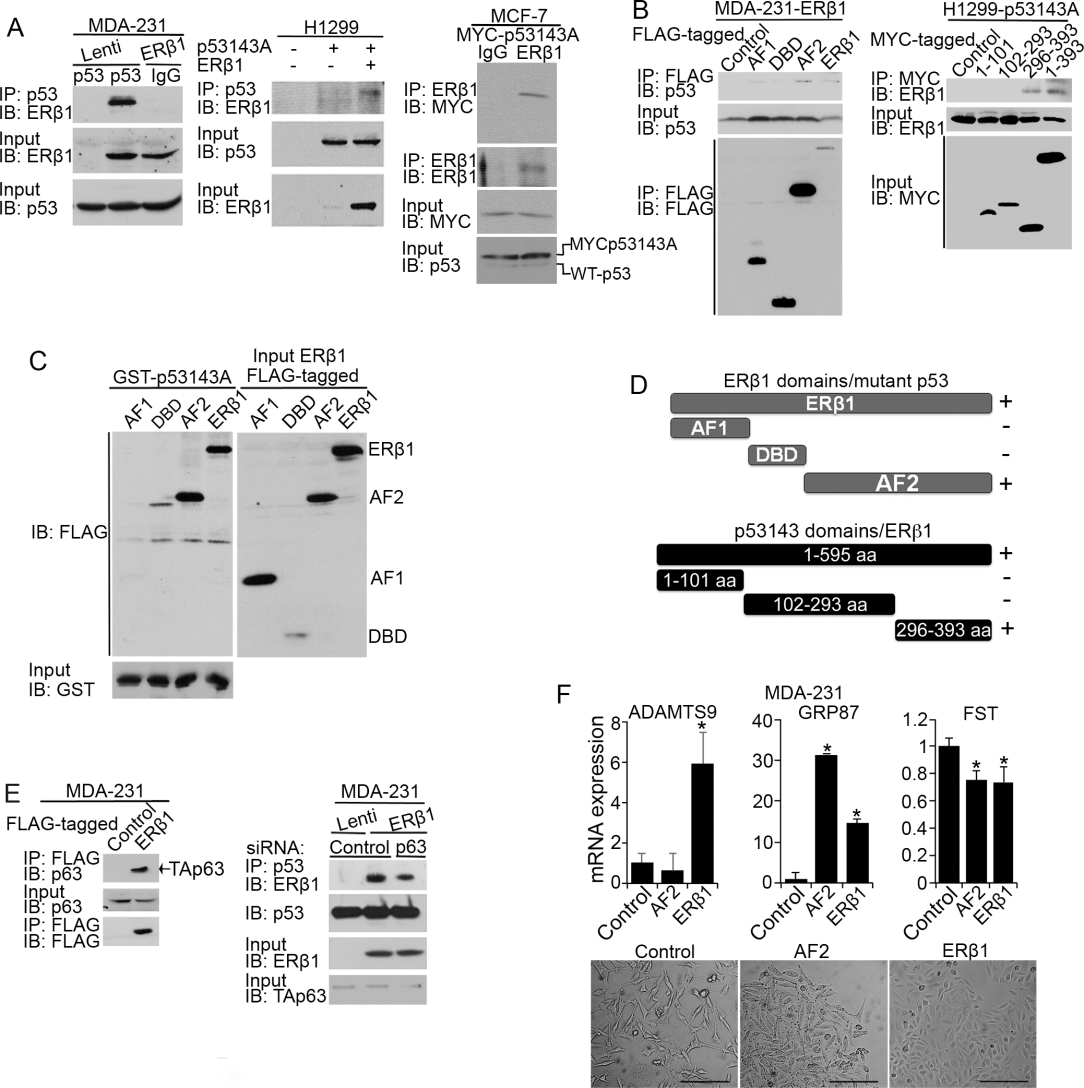
ERβ1 interacts with mutant p53 and p63 (**A**) Lysates from control and ERβ1-expressing MDA-MB-231 cells (left) or control, p53143A-, and p53143A/ERβ1-expressing H1299 cells (middle) were immunoprecipitated with anti-p53 antibody, followed by immunoblotting with ERβ1 antibody. Right: Lysates from MCF-7 cells transfected with MYC-p53143A were immunoprecipitated with anti-ERβ1 antibody or IgG, followed by immunoblotting with anti-MYC or anti-ERβ1 antibodies. The bottom panels are the input controls of cell lysates. (**B**) Left: Lysates from MDA-MB-231 cells stably expressing FLAG-tagged full-length ERβ1 or its activation function 1 (AF1), DNA-binding (DBD) and activation function 2 (AF2) domains were immunoprecipitated with anti-FLAG antibody, followed by immunoblotting with anti-p53 and anti-FLAG antibodies. Right: Lysates from H1299 cells stably co-transfected with empty vectors (control) or full-length ERβ1 together with MYC-tagged full-length mutant p53143A or its N-terminal (1–101 aa), DBD (102–293 aa) and C-terminal (296–393 aa) domains were immunoprecipitated with anti-MYC antibody followed by immunoblotting with anti-ERβ1 antibody. (**C**) GST-tagged p53V143A expressed in bacteria was used as bait protein to capture in pull-down assay *in vitro* translated FLAG-tagged full-length ERβ1 or its AF1, DBD and AF2 domains. (**D**) Schematic representation of ERβ1 truncations interacting with mutant p53 (top) and mutant p53 truncations interacting with full-length ERβ1 (bottom). (**E**) Left: Lysates from control and FLAG-tagged ERβ1-expressing MDA-MB-231 cells were immunoprecipitated with anti-FLAG antibody, followed by immunoblotting with anti-p63 and anti-FLAG antibodies. Right: Lysates from control and ERβ1-expressing MDA-MB-231 cells after transfection with control or p63 siRNA (#1) were immunoprecipitated with anti-p53 antibody, followed by immunoblotting with ERβ1 antibody. (**F**) mRNA levels of *ADAMTS9*, *GRP87* and *Follistatin* (FST) (top) and morphology (bottom) of MDA-MB-231 cells expressing full-length ERβ1 or its AF2 domain (scale bars, 200 μm).

A transcriptional cooperation between ERβ and p53 was previously associated with the binding of the proteins to their cognate response elements [22]. To examine whether an ERβ1-mutant p53 binding occurs, we performed co-immunoprecipitation (CoIP) experiments in lysates from p53280K-expressing MDA-MB-231 and p53143A-transfected H1299 cells. As shown in Figure 3A, an interaction between ERβ1 and p53 mutants was observed in both MDA-MB-231 and H1299 cells. In addition to transfected ERβ1, mutant p53 bound to endogenously expressed receptor in MCF-7 cells (Figure 3A, right). To define the interacting regions of the two proteins we examined a series of N-terminal, C-terminal and DBD deletion mutants of ERβ1 and p53143A. Analysis of MDA-MB-231 cells expressing ERβ1 domains and H1299 cells expressing p53143A truncations revealed that the C-terminus (296–393 aa) of mutant p53 and AF2 of ERβ1 are indispensable for their interaction (Figure 3B and Supplementary Figure 2). GST pull-down experiments with GST fusion mutant p53 expressed in bacteria and *in vitro* translated ERβ deletion mutants confirmed the AF2 of ERβ1 as the mutant p53 interacting region (Figure 3C and 3D). In addition to ERβ1, the C-terminus of mutant p53 was previously shown to bind to many other proteins and is required for the inhibition of p63 function and promotion of invasion [3]. Given that mutant p53 is tethered to specific chromatin regions through p63 [16], we examined whether ERβ1 binds to p63. As shown in Figure 3E, p63 interacted with ERβ1 and silencing of p63 in MDA-MB-231 cells decreased the association of ERβ1 with mutant p53. These findings suggest a role for p63 in the regulation of the ERβ1-mutant p53 interaction. Furthermore, we tested whether the AF2 of ERβ1 alone is able to suppress mutant p53 function. As shown in Figure 3F (top), the expression patterns of two of the mutant p53 target genes (*GRP87, Follistatin*) in AF2-expressing MDA-MB-231 cells were similar with those in cells expressing full length ERβ1. This was associated with the more epithelial-like morphology of AF2-expressing cells, suggesting that the AF2 is essential for the effect of ERβ1 on mutant p53 function (Figure 3F, bottom).

### p63 affects the regulation of mutant p53 function by ERβ1

**Figure 4:**
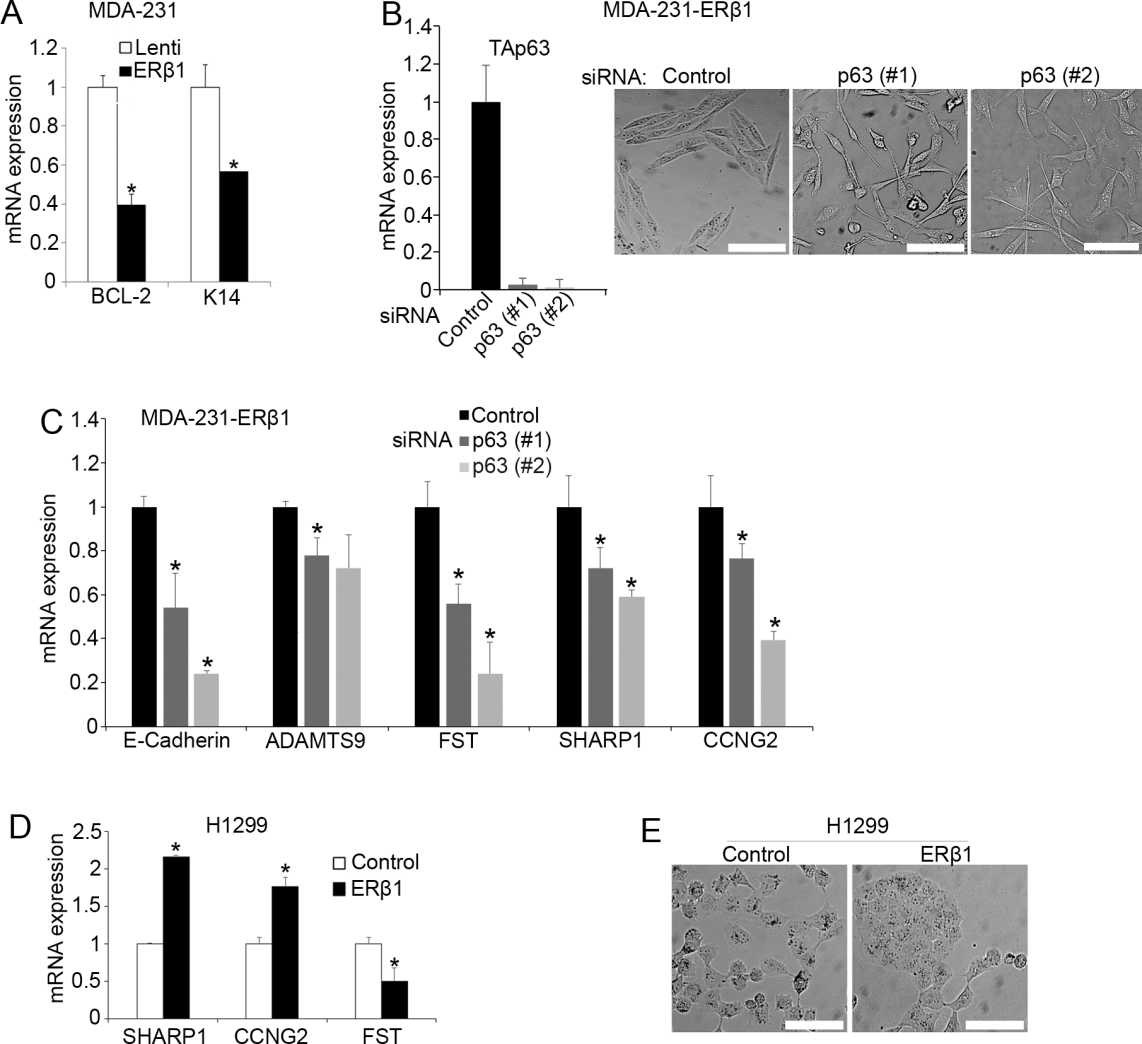
p63 affects the regulation of mutant p53 by ERβ1 (**A**) mRNA levels of the p63 target genes *K14* and *BCL-2* in control and ERβ1-expressing MDA-MB-231 cells. The graph shows the mean of three experiments; *P ≤ 0.05. (**B**) Levels of TAp63 and morphology of ERβ1-expressing MDA-MB-231 cells following transfection with control or two siRNAs targeting p63 (scale bars, 100 μm). (**C**) mRNA levels of E-cadherin and mutant p53 target genes in ERβ1-expressing MDA-MB-231 cells after transfection with p63 siRNA (*Follistatin*, FST). Values represent the mean ± S.D. of three experiments; *P ≤ 0.05. (**D**–**E**) mRNA levels of mutant p53 target genes and morphology of control and ERβ1-expressing H1299 cells (scale bars, 100 μm).

p53 mutants have been shown to promote invasion by preventing normal TAp63 function [10, 12, 28]. Given that ERβ1 inhibited both invasion and mutant p53 function in TNBC cells, we investigated whether activation of TAp63 is essential for the anti-invasive activity of ERβ1. We initially found that ERβ1 alters the expression of the p63 target genes K14 and BCL-2 in MDA-MB-231 cells that express both TA and ΔNp63 (Figures 4A and 1D) [37]. Importantly, transfection with siRNA that downregulates both p63 isoforms reversed to a significant extent the epithelial-like morphology and upregulation of E-cadherin in ERβ1-expressing MDA-MB-231 cells (Figure 4B and 4C and Supplementary Figure 3) [27]. In addition, knockdown of p63 significantly reversed the ERβ1-induced expression of the mutant p53 target genes *SHARP-1*, *ADAMTS9* and *CCNG2*. In contrast, the levels of *Follistatin* were further decreased which may reflect a different effect of TAp63 and ΔNp63 downregulation on the expression of some of the ERβ1/mutant p53-regulated genes (Figures 4C and 1A). These results suggest that p63 is involved in the mechanism employed by ERβ1 to inhibit the invasive phenotype of TNBC cells and are consistent with the regulation of mutant p53-ERβ1 interaction by p63 (Figure 3E). To examine whether ERβ1 regulates p63 target genes in a mutant p53-independent manner, we analyzed the expression of *SHARP-1*, *CCNG2* and *Follistatin* in p53 null H1299 cells that express endogenous p63 (Figure 1D). As shown in Figure 4D and 4E, upregulation of ERβ1 altered the expression of these genes and the morphology of cells in absence of p53, albeit to a lesser degree than in presence of mutant p53 suggesting that ERβ1 can directly act on p63.

### ERβ1 interferes with the regulatory elements of mutant p53/p63 target genes

**Figure 5:**
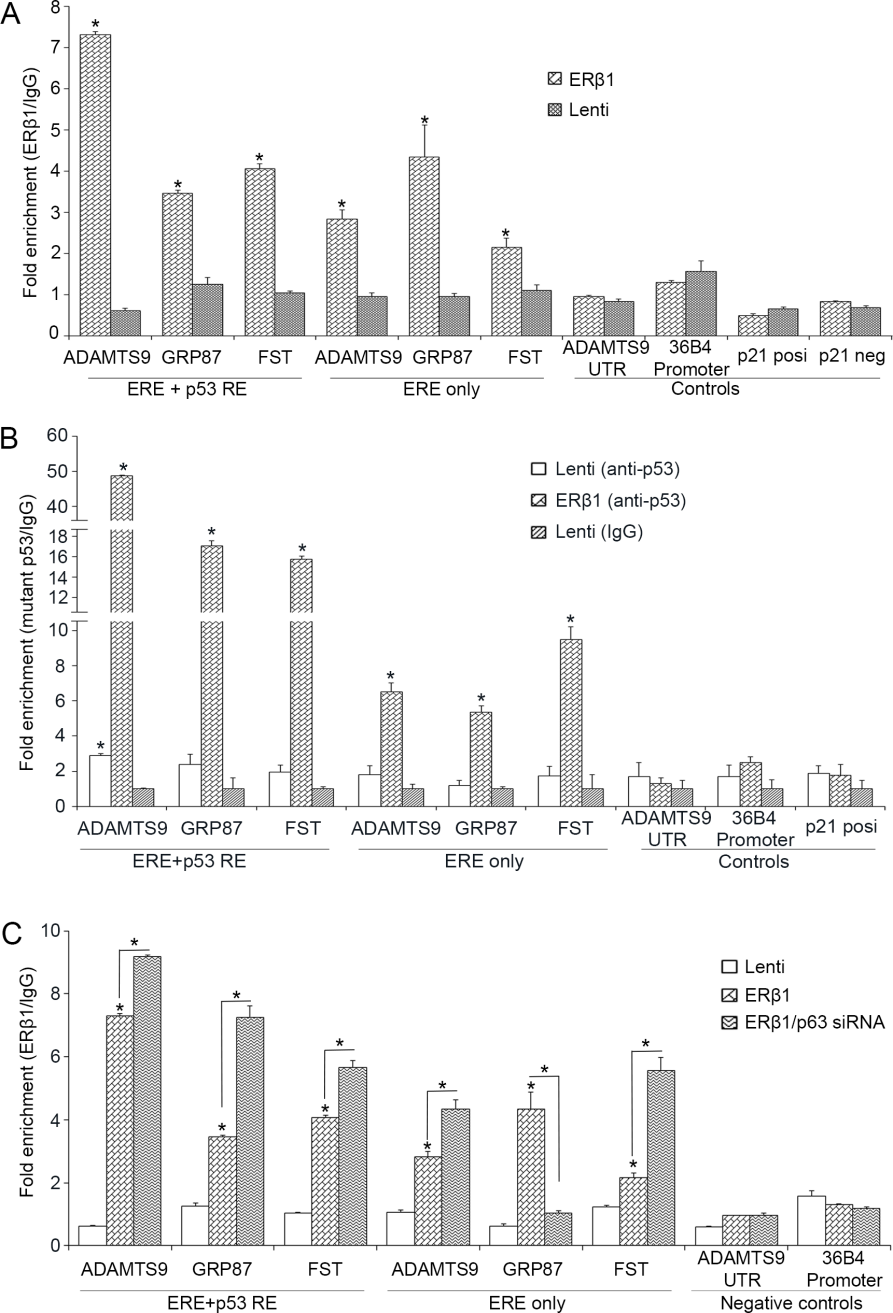
ERβ1 binds to promoters of mutant p53 target genes (**A**) Chromatin immunoprecipitation (ChIP) analysis in control and ERβ1-expressing MDA-MB-231 cells for the presence of ERβ1 at sites of mutant p53 target genes that contain both ERE and p53REs or exclusively ERE (*Follistatin*, *FST*). ERE/p53REs-negative sites from *36B4* promoter and downstream of the *ADAMTS9* gene and a 5′ p53RE from *p21* promoter that binds wild-type p53 were used as controls. Anti-FLAG antibody was used to immunoprecipitate ERβ1 and normal mouse IgG was used as experimental control. Fold enrichment of p53 target sequences was normalized to IgG ChIP. (**B**) ChIP analysis for binding of mutant p53 at sites of mutant p53 target genes in control and ERβ1-expressing MDA-MB-231 cells. (**C**) ChIP analysis for the presence of ERβ1 at sites of mutant p53 target genes in control and ERβ1-expressing MDA-MB-231 cells following transfection with control or p63 siRNA (#1). Fold enrichment of target sequences in ERβ1 precipitates was normalized to that of IgG precipitates. All graphs represent the mean ± SEM of three experiments; *P ≤ 0.05.

Given the binding of ERβ1 to both mutant p53 and p63, we investigated whether ERβ1 interacts with regulatory elements of mutant p53/p63 target genes. We performed chromatin-immunoprecipitation (ChIP) experiments in MDA-MB-231 cells to examine whether ERβ1 binds to sites that contain either both ERE and p53REs or exclusively ERE within the promoter region and close to the first exon of *ADAMTS9*, *GRP87* and *Follistatin* genes (Supplementary Figure 4). We also analyzed ERE/p53REs-negative sites from *36B4* promoter and downstream of the *ADAMTS9* gene as well as a 5′ p53RE from *CDKN1A* (*p21*) promoter that binds to wild-type p53 [38]. As shown in Figure 5A, a strong association of ERβ1 with ERE/p53REs-containing sites of *ADAMTS9*, *GRP87* and *Follistatin* was detected in ERβ1-expressing cells. The association of ERβ1 with the ERE-containing sites of the same genes was also induced in ERβ1-expressing cells, albeit at significantly lower degree in case of *ADAMTS9* and *Follistatin*. These results suggest that both direct DNA binding and interaction with mutant p53 may be necessary for ERβ1 to associate with promoters of mutant p53 target genes (Figure 5A).

To examine whether upregulation of ERβ1 alters the binding of mutant p53 to promoters of its target genes, we performed ChIP for p53 in control and ERβ1-expressing MDA-MB-231 cells. As shown in Figure 5B, in contrast to *p21* promoter, the association of mutant p53 with *ADAMTS9*, *GRP87* and *Follistatin* sites was dramatically induced in ERβ1-expressing cells. This suggests that ERβ1 may specifically increase the binding of mutant p53 to genes that are associated with metastasis. An enrichment of the mutant p53-bound sequences that contain exclusively ERE motifs was also observed in ERβ1-expressing cells, suggesting that mutant p53 may tether to specific DNA sequences through ERβ1 (Figure 5B).

To examine whether p63 alters the binding of ERβ1 to regulatory elements of mutant p53 target genes, we performed ChIP in ERβ1-expressing MDA-MB-231 cells following knockdown of p63. As shown in Figure 5C, the binding of ERβ1 to ERE/p53REs of *ADAMTS9*, *GRP87* and *Follistatin* promoters was induced by p63 downregulation. In contrast, p63 knockdown decreased the ERβ1 association with the ERE motif of *GRP87* promoter suggesting that p63 may regulate direct ERβ1-DNA binding (Figure 5C). Taken together, these results suggest an interaction of ERβ1 with promoters of mutant p53 target genes, which is regulated by mutant p53 and p63.

### Effects of ligands on ERβ1-mutant p53 interaction

**Figure 6:**
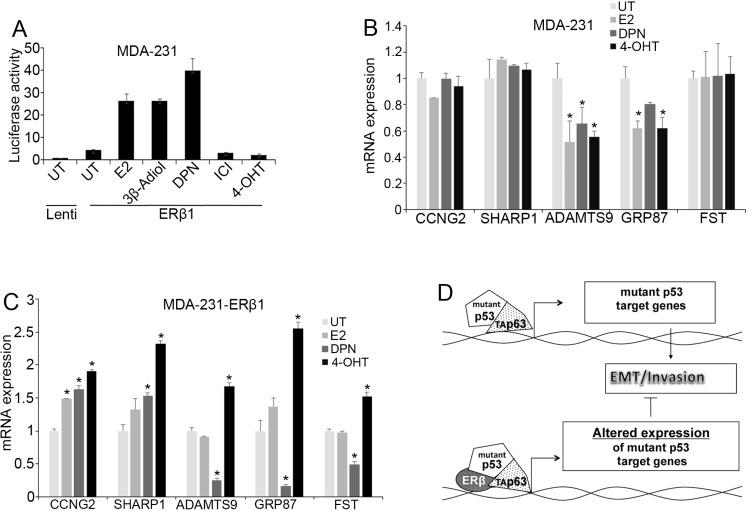
Effects of ER ligands on mutant p53 function (**A**) ERE-driven luciferase activity in control and ERβ1-expressing MDA-MB-231 cells after treatment with 10 nM 17β-estradiol (E2), Diarylpropionitrile (DPN), ICI 182780 (ICI) or 1 μM 5α-androstane-3β,17β-diol (3β-Adiol) or 4-hydroxytamoxifen (4-OHT) for 24 h. The graph indicates the mean ± S.D. of three experiments. (**B**–**C**) mRNA levels of mutant p53 target genes in control (B) and ERβ1-expressing (C) MDA-MB-231 cells after treatment with 10 nM E2, 10 nM DPN or 1 μM 4-OHT in DCC-containing media for 24 h (*Follistatin*, FST). Values represent the mean ±S.D. of three experiments; *P ≤ 0.05. (**D**) Proposed mechanism illustrating the regulation of mutant p53 function by ERβ1 in breast cancer cells. Mutant p53 alters the activity of transcription factors including p63. This results in a gene expression program that promotes cell invasion. By interacting with mutant p53, ERβ1 alters mutant p53-dependent gene expression impeding EMT and inhibiting invasion.

Upregulation of ERβ1 in cancer cells elicits tumor repressive actions in both ligand-dependent and -independent manner. Similarly, ligand-dependent and -independent target genes were identified in ERβ1-expresing cancer cells [27, 34, 39–41]. In this study, we investigated whether ligands that bind ERβ1 alter the function of mutant p53. The ERβ1 agonists 17β-estradiol (E2), 5α-androstane-3β,17β-diol (3β-Adiol) and Diarylpropionitrile (DPN) but not the ERα antagonist tamoxifen significantly increased the ERE-dependent transcription in ERβ1-expressing MDA-MB-231 cells (Figure 6A). In contrast, tamoxifen significantly increased the expression of all the mutant p53 target genes in ERβ1-expressing cells but not in control cells (Figure 6B and 6C). This may suggest that tamoxifen increases ERβ1 transcriptional responses when the receptor cooperates with other transcription factors. In addition to tamoxifen, the ERβ1-selective agonist DPN altered the expression of *SHARP-1*, *CCNG2* and *Follistatin* but not *ADAMTS9* and *GRP87* in the same mode as the upregulation of ERβ1 did (Figure 6B and 6C and Figure 1A and 1B). These results support the presence of a functional receptor in TNBC cells and the ability of ER ligands to regulate mutant p53 function.

## DISCUSSION

In the present study we examined whether inhibition of mutant p53 oncogenic function accounts for the decreased invasiveness of ERβ1-expressing TNBC cells. We found that ERβ1 upregulates mutant p53 target genes that are associated with normal phenotype and decreases the expression of pro-metastatic factors. This effect was observed in TNBC and other human cancer cell lines that harbor different tumor-associated p53 mutations, indicating the ability of the receptor to alter mutant p53-dependent transcription across different forms of mutant p53. Small divergence in the expression of some of the p53 target genes across ERβ1-expressing cell lines may be attributed to the different function of mutant p53 in cells with varying expression of TA and ΔNp63 and other cofactors.

We also examined whether an ERβ1-mutant p53 interaction is required to modify the mutant p53 function. A strong binding of ERβ1 to different p53 mutants was observed in triple-negative and other types of cancer cells. Further analysis revealed that the AF2 domain of ERβ1 interacts with the C-terminus of mutant p53. This region of mutant p53 binds to many other proteins [42, 43] and inhibits the function of p63 [18]. Our experiments showed that ERβ1 interacts with p63. This suggests that ERβ1 may repress mutant p53 function through its recruitment to mutant p53-p63 complexes at promoters of mutant p53/p63 target genes (Figure 6D). ChIP assays confirmed the association of ERβ1 with such promoters through both direct binding and tethering mechanisms. The direct binding is also supported by the ability of ERβ1 to regulate the same genes in absence of p53. Importantly, ERβ1 was also found to increase the association of mutant p53 with p53REs. Wild-type p53 binds the same promoters [16] and mutant p53 shows impaired binding to standard p53REs [3]. Thus, our findings may imply that the ERβ1-mutant p53 interaction readjusts the folding of mutant protein into a wild-type conformation that leads to reactivation of wild-type function. Notably, binding of mutant p53 to ERE motifs of the same promoters in the presence of ERβ1 suggests that mutant p53 may regulate ERβ1 transcriptional activity. This model of mutant p53 function was previously described with other transcription factors [3].

In this study, we propose that the anti-invasive activity of ERβ1 in breast and perhaps in other types of cancer cells may correlate with its ability to interact with oncogenic mutant p53. The relevance of this interaction to breast cancer may depend on the expression of the two proteins in tumor cells. Although the expression of ERβ has been suggested to decline in invasive carcinomas [44–46], a significant of number of breast tumors including TNBCs express the receptor [19, 21, 47, 48]. In this study, lower levels of ERβ1 were found to interact with mutant p53 in TNBC cells compared with those of ERα that interact with wild-type p53 in ERα-positive breast cancer cells [24, 25]. Importantly, the ERβ1-mutant p53 binding was also detected in breast cancer cells that naturally express the receptor. The functionality of this interaction is supported by the ligand-dependent inhibition of mutant p53 function in ERβ1-expressing cells. This is consistent with the previously identified ligand-dependent ERβ1 target genes in TNBC cells [40]. Some of the ER ligands that act as ERα antagonists have been reported to induce ERβ1-mediated tumor repressive actions [40, 49–51]. The inhibitory effect of tamoxifen on mutant p53 function in the presence of ERβ1 suggests that patients whose p53-defective breast tumors are positive for the receptor may benefit from treatment with ER ligands. This effect may also explain the previously reported association of ERβ1 with better survival in patients treated with tamoxifen [19]. Further understanding of the mechanism that regulates the occupancy of both proteins at promoters of common target genes should establish ERβ1 as an important regulator of mutant p53 oncogenic function in breast cancer.

## MATERIALS AND METHODS

### Cells, reagents and constructs

All cell lines were obtained from ATCC and cultured in RPMI-1460 or Dulbecco's Modified Eagle Medium media supplemented with 10% fetal bovine serum (FBS) at 37°C in 5% CO_2_. Phenol red-free medium supplemented with 0.5–1% dextran-coated charcoal (DCC)-treated FBS was used in ligand treatments. For stable expression of ERβ1, cells were infected with pLenti6/V5-FLAG-ERβ1 construct as described previously [27]. The pINDUCER20-FLAG-ERβ1 plasmid was transduced into cells and gradual ERβ1 expression was achieved following incubation of G418-selected cells with different concentrations of doxycycline (dox) for 24 hours (h) [52]. Cells were single- or co-transfected with the following plasmids: pIRESneo3, pIRESpuro3 (Clontech), pIRESneo-FLAG-ERβ1, pIRESpuro-FLAG-p53V143A, pIRESpuro-FLAG-p53V175H and pcDNA3-MYC-p53V143A. The cDNA of p53V175H and p53V143A were PCR-amplified from pCMV-Neo-Bam-p53V175H and -p53V143A plasmids (Addgene, plasmids #16436 and 16435). FLAG-tagged ERβ1 domains [AF1 (amino acids: 1–150), DBD (145–220), and AF2 (211–530)] were cloned into a pIRESneo3 vector and MYC-tagged p53V143A domains [N-terminus (1–101), DBD (102–293) and C-terminus (296–393)] into a pcDNA3 plasmid. The pGEX4T-1 plasmid was used for the bacterial expression of GST-tagged proteins. Cells were transfected twice with p63-specifc siRNAs from Invitrogen, target sequences: 1# 5′-ATTCCATGGTCGTGTGAGACAGAAG-3′ and 2# 5′-AACTTAAGCGCCGAGTCGAGTACCA-3′. An siRNA-targeting luciferase was used as control (Cat. No. 12935–146; Invitrogen). For p53 knockdown, cells were transfected twice with the pLKO-p53-shRNA-427 or scramble shRNA control plasmids (Addgene, plasmids #25636 and 1864). All transfections were performed using Lipofectamine 2000 (Invitrogen) as previously described [27].

### RNA extraction, real-time quantitative reverse transcription (RT) PCR

mRNA was isolated using the Aurum^™^ Total RNA Mini Kit (Biorad). RNA was reversed transcribed to cDNA using the iScript^™^ cDNA Synthesis Kit (Biorad). Real-time PCR was performed using the iTaq^™^ Universal SYBr Green Supermix (Bio-Rad). All quantitative data were normalized to *36b4*. Primer sequences for the real-time PCR experiments are listed in Supplementary Table 1.

### Luciferase assays

For assessing ERE-dependent transcriptional activity, cells were maintained in phenol red free DCC-containing media for 48 h, transiently co-transfected with 3-ERE-TATA-LUC reporter plasmid and a plasmid expressing β-galactosidase and incubated in the presence of ligands for 24 h. Luciferase activity was measured as previously described [41].

### Co-immunoprecipitation and immunoblotting

Cells were plated at a density of 5 × 10^5^−10^6^ per 10-cm dish and lysed in immunoprecipitation (IP) buffer containing 50 mM Hepes pH 7.4, 150 mM NaCl, 1 mM EDTA, 0.8 mM EGTA, 1 mM NP-40, 1 mM Glycerol, 2 mM PMSF, 1 mM Na_3_V0_4_, 50 mM NaF, 1% protease inhibitor cocktail (Roche) and 1% phosphatase inhibitor mixture (Sigma). Supernatants were clarified by centrifugation and incubated with specific antibodies overnight and A/G agarose beads (Santa Cruz) or protein G magnetic beads (Biorad) for 3 h. Immunoprecipitates were subjected to SDS-PAGE and transferred onto nitrocellulose membrane (Amersham Biosciences). Membranes were probed with the primary antibodies overnight at 4°C and proteins were visualized using ECL detection kit (Amersham Biosciences) as previously described [39]. ERβ1 and its truncations were immunoprecipitated with anti-FLAG M2 affinity gel (Sigma) and the precipitates were immunoblotted with a rabbit antibody against p53 (Cell signaling). For the reverse experiments, antibodies against p53 (DO-1 or Pab 240; Santa Cruz) were used for immunoprecipitation and ERβ1 antibodies (rabbit polyclonal, Millipore or 14C8, GeneTex) for immunoblotting. MYC-tagged p53V143A and its truncations were immunoprecipitated with anti-MYC antibody (Cell signaling). Antibodies against p63 and ΔNp63 were from Abcam (BC4A4) and Biolegend, respectively. Antibodies against SHARP-1, Follistatin were from Santa Cruz and that of CCNG2 from Abcam.

### Chromatin immunoprecipitation

Protein-DNA complexes were crosslinked with 1% formaldehyde for 10 min at room temperature (RT). Cells were harvested and cell suspensions were centrifuged. For nuclei purification, cell pellets were washed (PBS, NCP1 and NCP2 buffers) and incubated in lysis buffer (10 mM EDTA, 20 mM Tris-HCl pH 8.1, 0.5 mM Empigen, 1 mM SDS and 1% protease inhibitor mixture) for 10 min at RT. Chromatin in the nuclear extract was sheared by sonication, clarified by centrifugation and the supernatant was used for immunoprecipitation as previously described [53]. Chromatin-bound mutant p53 was incubated with an anti-p53 antibody or IgG and precipitated with protein G magnetic beads. Anti-FLAG M2 magnetic beads (Sigma) were used for the precipitation of the chromatin-bound ERβ1. Protein-DNA complexes were eluted and decrosslinked at 70°C overnight and DNA enrichment was measured by real-time PCR using promoter-specific primers (Supplementary Table 1).

### Protein expression and GST pull-down assay

GST-tagged proteins were produced as described previously [54]. Briefly, isopropyl β-D-1-thiogalactopyranoside (IPTG) was added to the culture of transformed bacterial cells with pGEX4T-1-p53143A recombinant plasmid to induce expression of GST-tagged protein. Protein interaction was assessed using a GST pull-down kit (Thermo Scientific) according to the manufacturer's protocol. GST-tagged proteins were used as bait to capture the full length or ERβ1 truncations that were expressed *in vitro* using the TnT^®^ Quick Coupled Transcription/Translation System (Promega). Glutathione agarose beads were used to precipitate the GST-tagged protein complexes overnight. The interacting proteins were eluted and subjected for analysis to SDS-PAGE and immunoblotting.

### Migration and invasion assays

In the wound-healing assay, the cell monolayer was scratched with a pipette tip to form the wound. Images of the wound were taken when the cells were scraped and 12 h later, and the area of cell migration in the wound was measured. Invasion assay was performed as described previously [27]. Cells were plated in matrigel-coated 6.5 mm Transwell champers (BD Biosciences). 6 h later, the cells that had been invaded through the filter and attached to its bottom surface were stained with crystal-violet and counted in five independent fields in each Transwell.

### Statistical analysis

Student's *t*-test and ANOVA were used for statistical analysis. Statistical significance was obtained when *p*-value ≤ 0.05.

## SUPPLEMENTARY MATERIALS FIGURES AND TABLE


