# Supplementary material for: ERβ decreases the invasiveness of triple-negative breast cancer cells by regulating mutant p53 oncogenic function
Source: Oncotarget. 2016 Feb 10;7(12):13599–611. doi: 10.18632/oncotarget.7300 (PMC4924664; doi:10.18632/oncotarget.7300)
Supplement: Supplementary file 1 [file oncotarget-07-13599-s001.pdf]

ERβ decreases the invasiveness of triple-negative breast cancer cells by regulating mutant p53 oncogenic function

Supplementary Materials

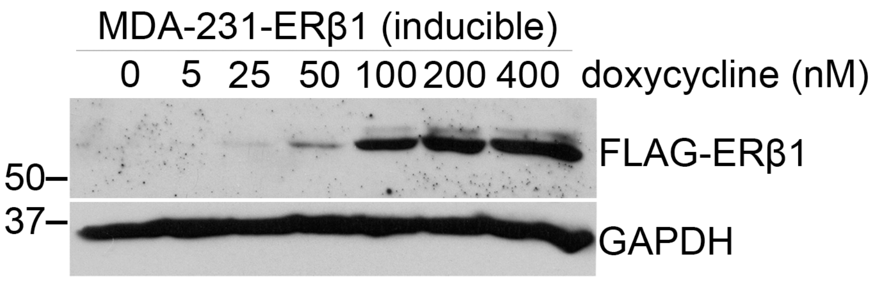

Supplementary Figure S1: Gradual expression of ERβ1. MDA-MB-231 cells were infected with lentivirus containing the pINDUCER20-FLAG-ERβ1 recombinant plasmid. Gradual ERβ1 expression was achieved following incubation of G418-selected cells with different concentrations of doxycycline (dox) for 24 h. ERβ1 expression was analyzed by immunoblotting.

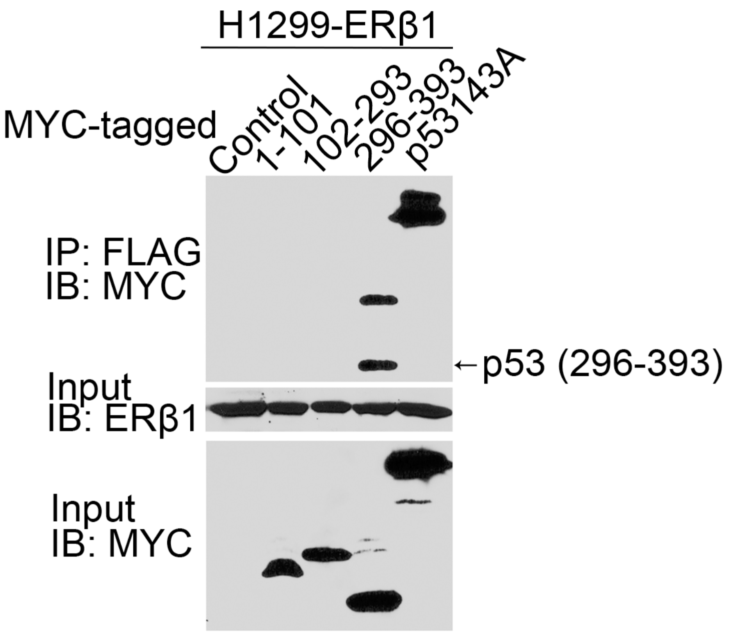

Supplementary Figure S2: ERβ1 interacts with mutant p53. Lysates from H1299 cells stably co-transfected with empty vectors (control) or FLAG-tagged ERβ1 together with MYC-tagged full-length mutant p53143A or its N-terminal (1–101 aa), DBD (102–293 aa) and C-terminal (296–393 aa) domains were immunoprecipitated with anti-FLAG antibody followed by immunoblotting with anti-MYC antibody. The bottom panels are the input controls of cell lysates.

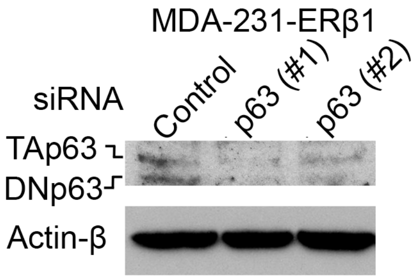

Supplementary Figure S3: siRNA-mediated downregulation of p63. Protein levels of TAp63 and ΔNp63 in ERβ1-expressing MDA-MB-231 cells after transfection with control or two siRNAs targeting p63.

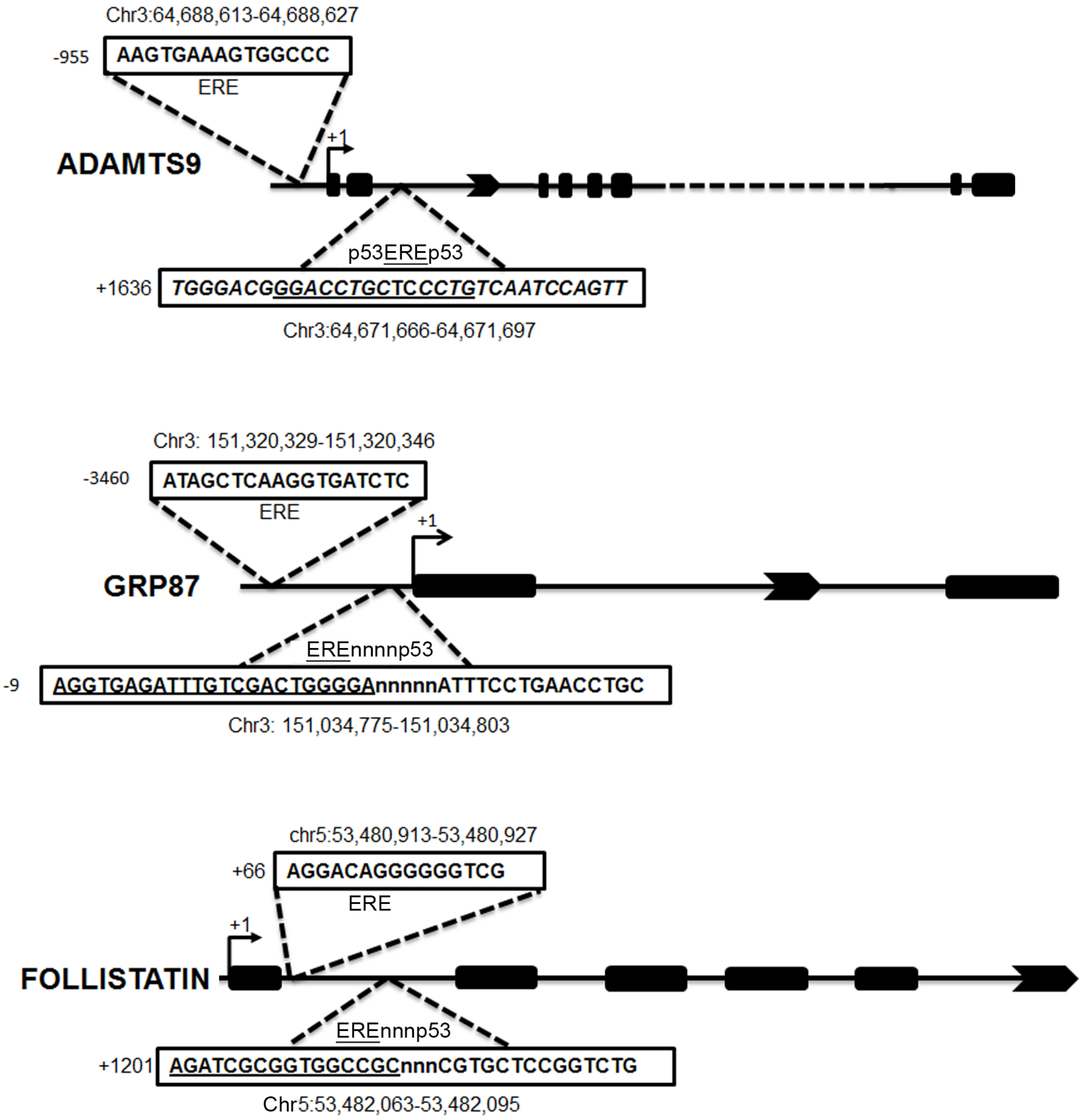

Supplementary Figure S4: Binding sites of ERβ1 on mutant p53 target genes. *ADAMTS9, GRP87* and *FOLLISTATIN* genomic sequences were extracted from University of California Santa Cruz (UCSC) genome browser. GPminer and DBTSS were used to identify potential transcription start sites. The start sites of first exons were labeled as +1. Jaspar database was used to find potential EREs and p53/p63REs. Their chromosomal locations and relative distances to the first exons are indicated. Schemes of *GRP87* and *ADAMTS9* have been adjusted to illustrate sequences of the negative strands. Values are given with an error of ± 1.

Supplementary Table S1: Oligonucleotides used in ChiP (in 5′ to 3′ direction)

ERE + P53 binding motifs	**ADAMTS9-prom FW**	CAATTCAAAGCGTCCGGGG
	**ADAMTS9-prom RV**	TTATTCACGCGCCAGGAACC
	**GRP87-prom FW**	GT GAGATTT GT CG ACTGGGGA
	**GRP87-prom RV**	TCTAAGCTCCAGCCCTCTGT
	**FST-prom FW**	GCAGCCCTCGGAGATTTCG
	**FST-prom RV**	CCGTTTTGCAATCCGCCAGA
ERE only	**ADAMTS9-ERE FW**	CT CTT CCGT CCCCATCTCTTG
	**ADAMTS9-ERE RV**	GT GCC AAAT CT CTT CCCGAAT G
	**GRP87-ERE FW**	CCTT GTTGCATGTTCCAGCC
	**GRP87-ERE RV**	TGCACATATAAAGGGCGTCA
	**FST-ERE FW**	TT CAT GGAGGACCGCAGTG
	**FST-ERE RV**	AGACCACAGAAAAGTCGACCC
Controls	**ADAMTS9 dwst-UTR FW**	GAAACTGAGT GGATGCCTGC
	**ADAMTS9 dwst-UTR RV**	CAATATCAAGGGGCCTGGGA
	**36B4-prom FW**	GTTGAGCAACATTAGGCAAGC
	**36B4-prom RV**	ACAGGCACCTTTTTAAAATCTCATT
	**P21-FW**	CTGGACT GGGCACT CTT GT C
	**P21-RV**	CTCCTACCATCCCCTTCCTC

Oligonucleotides used in real-time PCR (in 5′ to 3′ direction)

**TAp63-FW 1**	AAGATGGTGCGACAAACAAG
**TAp63-RV 1**	AGAGAGCATCGAAGGTGGAG
**TAp63-FW 2**	CCTGACCCTTACATCCAGCG
**TAp63-RV 2**	CGGTTCATCCCTCCAACACA
**kl4-FW**	GCAGTCATCCAGAGATGTGAC
**kl4-RV**	GCCTCAGTTCTTGGTGCGA
**BCL-2-FW**	GATAACGGAGGCTGGGATGC
**BCL-2-RV**	TCACTTGTGGCCCAGATAGG
**SHARP1-FW**	CGTCTTTGGAGTTGACATGG
**SHARP1-RV**	GGGCAGCTTTGAGAACTAGC
**CCNG2-FW**	TGGACAGGTTCTTGGCTCTT
**CCNG2-RV**	GATGGAATATTGCAGTCTTCTTCA
**ADAMTS9-FW**	GAACGCGACGGAGCATTAAC
**ADAMTS9-RV**	TAGAAACTGCTGGCCGAAGG
**GRP87-FW**	TCCTGACACGCATCTTTGCT
**GRP87-RV**	CCGTGCAGCTCGTTATTTGG
**FST-FW**	CAATGCCACTTATGCCAGCG
**FST-RV**	TCGGTGTCTTCCGAAATGGAG
